# Genomic technologies—from tools to therapies

**DOI:** 10.1186/s13073-017-0462-9

**Published:** 2017-07-31

**Authors:** Andreia Cunha

**Affiliations:** Genome Medicine, BioMed Central, London, UK

We are very pleased to invite you to read the first articles in our special issue featuring the development and clinical application of genomic technologies. Our goal was to capture the recent advances in a broad range of technologies used to analyze or manipulate genomic information with the potential to aid in understanding, preventing, diagnosing, and treating disease [1]. The past few years have seen extraordinary developments in areas such as high-throughput sequencing, big-data analysis and storage, genome engineering, and gene therapy. It is now clear that genomic technologies will make a real impact in the clinic, and, although their full potential is still far from being met, areas where transformative applications have been made already include oncology and genetic disease diagnostics.

A particularly active area has been the development of tools for tumor DNA sequencing and analysis. It is now possible to perform high-throughput sequencing of tumor samples and identify the mutations in a patient’s tumor, thus allowing a precise diagnosis and selection of the most appropriate therapy. A common problem in the accurate identification of somatic mutations (genetic changes not present in germline cells) in tumors is the absence of matched normal tissue. To counter this, Lincoln Stein and colleagues have developed ISOWN, software that uses supervised machine learning combined with external databases to identify, with high accuracy, somatic mutations in next-generation sequencing data of tumor samples in the absence of normal samples [2]. ISOWN might be useful in situations where normal tissue was not collected, the patient consent does not allow for its collection, or in retrospective studies.

Another challenging area in tumor sequencing is the identification of genomic rearrangements (gross DNA alterations of chromosomes or large chromosomal regions). Short-read whole-genome sequencing is currently the gold-standard approach, although its accuracy is suboptimal and it is expensive and labor intensive. A proof-of-principle study by Hanlee Ji and colleagues proposes novel methods to resolve genomic rearrangements that drive tumorigenesis using barcode-linked read sequencing of whole genomes [3]. Short-read sequencing is also not an optimal approach to determine trinucleotide repeat counts; this is of paramount importance because trinucleotide-repeat diseases, such as Huntington’s disease, will often manifest only after a certain threshold trinucleotide count, and the number of repeats may influence disease severity. Developments in this area of research have been made by Kai Wang and colleagues [4], where they present RepeatHMM, software that can effectively and efficiently quantify repeat counts from long-read sequencing.

Understanding of Mendelian diseases and complex diseases is also improving, and recently there have been notable advances in neuropsychiatric genomics. Identifying genes involved in these complex disorders should allow a better understanding of disease and improve diagnostics and therapy. Melanie Bahlo and colleagues have developed a web-application, called brain-coX, that focuses on gene prioritization and exploration of gene networks for diseases that originate in human brain tissue in order to identify candidates potentially involved in neurological disorders [5]. Such tools should inevitably draw us closer to mapping the biological basis of psychiatric diseases.

Disease etiology has been linked to microbiome composition, with perhaps the best characterized example so far being the causal link between high levels of *Fusobacterim nucleatum* in the gut and development of colorectal cancer [6, 7]. In a Comment, Ramnik Xavier and Andrew Tolonen discuss state-of the-art microbial single-cell sequencing technologies and their potential to help characterize the microbiome at the cellular level and understand its role in immunity and disease [8].

Exciting advances are also being observed in genomic technologies to devise better therapies. A valuable tool to identify vulnerabilities in cancer cells that can be targeted for therapy is functional genomic screening with short hairpin RNA (shRNA). However, this field has been plagued by low reproducibility owing to off-target effects. Tero Aittokallio and colleagues perform a systematic comparison of the consistency of two genome-wide shRNA screening datasets and propose a proof-of-principle approach to reduce noise in genome-wide shRNA screens to make them more consistent [9].

Proteogenomics is another approach to uncover druggable targets in cancer that is showing promise as it can identify germline and somatic mutations within crucial cancer genes missed by genomics and transcriptomics. Thomas Kislinger and colleagues perform a systematic investigation of how the choice of database in a proteogenomics study can affect the results and suggest a new strategy to assess the variant peptides identified and their potential impact on cancer biology [10].

The RNA therapeutics field is gaining momentum, with many new therapies reaching clinical trials and the first therapies gaining FDA approval. The main challenges with stability and immunogenicity seem to have been tackled, and delivery and safety are now in the spotlight. In a Review, Daniel Anderson and colleagues discuss recent advances in the delivery of RNA-based therapeutics, such as CRISPR-Cas9 genome-editing technology in research and clinical applications [11].

The large amounts of data generated by the ubiquitous use of sequencing technologies pose challenges to data storage. Cloud computing offers a solution that allows for data to be shared through potential collaboration, but there are regulatory hurdles that must be overcome. In an Opinion article, Jan Korbel and colleagues discuss the regulatory landscape facing cloud computing of patient data for genetics and genomics research both in Europe and in the international context and propose that a federated and hybrid cloud model could be a viable way forward [12].

With the reduction in costs for sequencing technologies expected to continue, more countries will be able to adopt these tools for applications in clinical research and healthcare, with more people able to benefit from data-driven and tailored treatments. *Genome Medicine* has been at the forefront of communicating advances in the development of genomic technologies with translational potential [13, 14] and demonstrating their clinical application [15]. Looking forward, we are committed to continuing to cover outstanding work in this field and, in particular, the latest in emerging areas.

We hope that you will enjoy this carefully curated collection of articles that showcases some of the most exciting current areas of the genomic technologies field. We will be adding more content over the next few months, so watch this space!
